# The “One-to-Many” Survival Analysis to Evaluate a New Treatment in Comparison With Therapeutic Alternatives Based on Reconstructed Patient Data: Enfortumab Vedotin Versus Standard of Care in Advanced or Metastatic Urothelial Carcinoma

**DOI:** 10.7759/cureus.28369

**Published:** 2022-08-25

**Authors:** Andrea Messori, Melania Rivano, Luca Cancanelli, Vera Damuzzo, Andrea Ossato, Marco Chiumente, Daniele Mengato

**Affiliations:** 1 Health Technology Assessment (HTA) Unit, Regione Toscana, Firenze, ITA; 2 Clinical Oncology Pharmacy Department, Armando (A) Businco Hospital, Cagliari, ITA; 3 Hospital Pharmacy Department, Azienda Ulss 2 Marca Trevigiana, Treviso, ITA; 4 Department of Pharmaceutical and Pharmacological Sciences, University of Padova, Padova, ITA; 5 Department of Pharmaceutical and Pharmacological Sciences, University of Padua, Padova, ITA; 6 Scientific Direction, Italian Society for Clinical Pharmacy and Therapeutics, Milano, ITA; 7 Hospital Pharmacy Department, Azienda Ospedale Università di Padova, Padova, ITA

**Keywords:** reconstruction of patient-level data, indirect comparisons, survival analysis, artificial intelligence, metastatic urothelial carcinoma

## Abstract

Objective

This paper presents a preliminary experience based on the "one-to-many" approach of the Shiny method. Numerous (or "many") treatments for advanced or metastatic urothelial carcinoma have recently been reviewed. More recently, "one" potentially innovative treatment has been made available. Our analysis was aimed at assessing the benefits of the new treatment in comparison with the alternatives developed previously.

Materials and methods

The Shiny method was employed to reconstruct patient-level survival data. This information allowed us to compare the Kaplan-Meier (KM) curves of five treatments previously available (i.e., pembrolizumab, nivolumab, atezolizumab, vinflunine, and standard chemotherapy) with the potentially innovative agent represented by enfortumab vedotin. Overall survival was evaluated for each agent. Statistical tests to assess head-to-head indirect comparisons were performed through standard survival analysis. The hazard ratio (HR) was the main parameter.

Results

In ranking the efficacy across these agents, enfortumab vedotin was first, followed by immune checkpoint inhibitors (ICIs). Standard chemotherapy and vinflunine were the least effective. The remarkable survival results of enfortumab were, to some extent, influenced by the slightly better prognosis of the population enrolled in the enfortumab trial in comparison with patients enrolled in the three ICI trials.

Conclusions

The experience described herein shows that, when a potential innovative treatment (enfortumab vedotin) is developed in an already investigated area (metastatic urothelial cancer), the Shiny method can be applied according to the "one-to-many" approach. This allows us to quickly assess the place in therapy of the new treatment (the "one") and to evaluate whether the new treatment determines a relevant incremental benefit in comparison with previous treatments (the "many").

## Introduction

The Shiny method [1,2] has emerged as a simple but powerful tool to perform indirect comparisons on the basis of survival data. This method represents an application of artificial intelligence in the reconstruction and interpretation of Kaplan-Meier (KM) time-to-event curves. The curves of overall survival (OS) or progression-free survival (PFS) are the most typical materials subjected to the Shiny method. In operational terms, each analysis based on the Shiny method processes one or more survival curves and generates the corresponding database of individual patient data (denoted as "reconstructed patient-level data" or "reconstructed individual patient data"). Hence, a simulated, hypothetical population of reconstructed patients is generated that includes as many patient groups as the number of treatments under examination. This population of simulated patients is the basis for performing multiple indirect comparisons across different treatments. Finally, an overall multi-treatment KM graph can be plotted to summarize, in comparative terms, the current evidence on the topic concerned. The main prerequisite for performing the Shiny analysis is that the same time-to-event endpoint (e.g., OS or PFS) has been employed in all the trials included in the comparison. Numerous experiences with the Shiny method have already been reported [3-14]; anti-cancer treatments are the most suitable area for this analysis [3-13].

In the present paper, we report a preliminary experience based on the Shiny method that examines the main treatments for advanced or metastatic urothelial carcinoma. In particular, the "one-to-many" approach of the Shiny method has been applied to evaluate the potential benefits of a new treatment (enfortumab vedotin) in comparison with multiple therapeutic alternatives (various regimens of chemotherapy and/or immunotherapy). The most typical framework in which the "one-to-many" approach can be applied is when comparing a new treatment proposed in a very recent trial with a certain number of well-established treatments aimed at the same disease condition.

## Materials and methods

The characteristics of the Shiny method have previously been described in detail [1,2]. It should be stressed that the reconstruction of patient-level data can have two different purposes: first, reconstructed individual patient data are estimated to determine the restricted mean survival time (RMST) [15]; second, reconstructed individual patient data are estimated to perform indirect comparisons between treatments and to generate a multi-treatment KM graph [3-14]. This second application of the Shiny method is increasingly considered the most important one. Irrespective of the purpose of the analysis, the reconstruction of patient-level data implies two subsequent phases: a first phase in which the graph of the KM curve is digitized (by using a standard tool such as Webplotdigitizer or other similar software), and a second phase in which the digitized curves are analyzed to generate individual patient data. This latter phase, which is much more complex than the first, is typically handled through the IPDfromKM subroutine [1], even though other tools have been used before 2021 [16]. The IPDfromKM package belongs to the area of artificial intelligence.

The present analysis focused on the treatments for advanced urothelial cancer. The main agents in this area were assumed to be those reported by Rivano et al. [17] (namely: pembrolizumab, nivolumab, atezolizumab, vinflunine, and standard chemotherapy). The trials analyzed by Rivano et al. [17] were those included in a previous network meta-analysis; one more trial was identified through a literature update. These trials enrolled patients with platinum-treated locally advanced or metastatic urothelial carcinoma. In terms of technical definitions, the trials on pembrolizumab, nivolumab, atezolizumab, vinflunine, and standard chemotherapy represent the "many" component of the "one-to-many" approach. The innovative treatment to be compared with previous treatments is represented by enfortumab vedotin. This agent has been reported in a trial by Powles et al. [18]. Enfortumab vedotin was therefore the "one" component in the "one-to-many" approach. Finally, regarding the "many" component, in the paper by Rivano et al., there was also a group given the best supportive care, which however was excluded from the present analysis owing to its insufficient effectiveness.

In operational terms, the analysis described herein was divided into two parts. In part 1, the reconstructed patient-level data were determined for the following five "previously available" treatments (i.e., pembrolizumab, nivolumab, atezolizumab, vinflunine, and standard chemotherapy); then, a first set of KM curves including these five treatments was generated. Thereafter, in part 2, we reconstructed patient-level data also for the trial by Powles et al. [18]. In more detail, the group treated with enfortumab vedotin (and also the group of controls given standard chemotherapy in Powles' trial) were added to the analysis generated in part 1. In this way, the second set of KM curves was generated that included pembrolizumab, nivolumab, atezolizumab, vinflunine, standard chemotherapy, and enfortumab vedotin. This graph was the final result of our analysis.

Regarding statistical analysis, OS was the endpoint in both parts 1 and 2. Statistical tests to assess head-to-head indirect comparison were performed through standard survival analysis. The hazard ratio (HR) was the main parameter. Finally, to assess the heterogeneity [14] across the trials, in the final analysis, the control groups of the trials on pembrolizumab, atezolizumab, and enfortumab vedotin were compared with one another. The trial on nivolumab did not include a control group. These three control groups had received standard chemotherapy as a common treatment.

## Results

**Figure 1:**
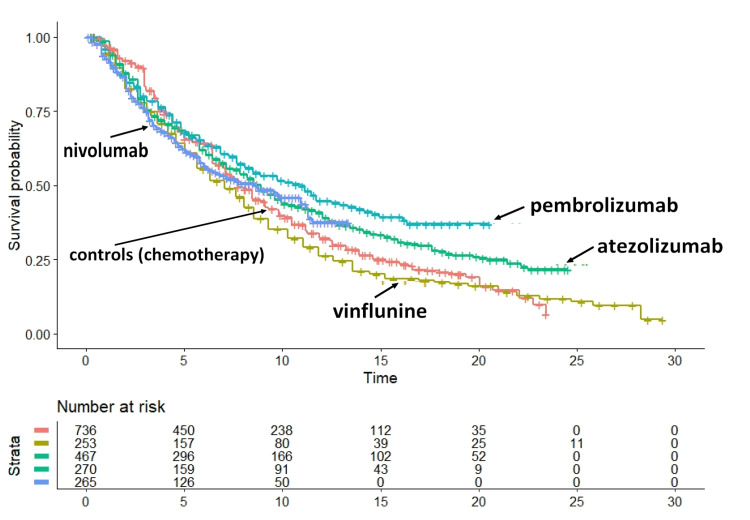
Analysis of five treatments previously proposed for advanced or metastatic urothelial cancer. This clinical material has been published by Rivano et al. [17]. Endpoint, overall survival. Time expressed in months.

Figure 1 shows the graph of the first set of KM curves, including pembrolizumab, nivolumab, atezolizumab, vinflunine, and standard chemotherapy (i.e., the so-called "previous treatments").

**Figure 2:**
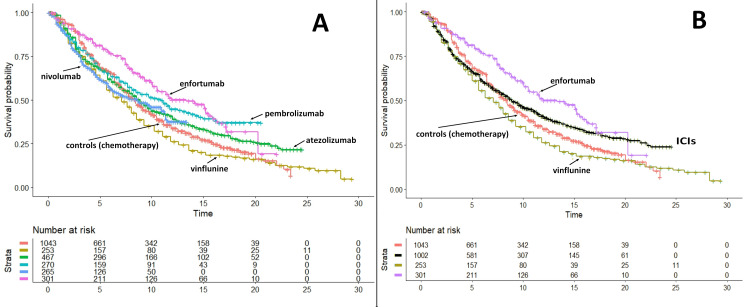
Results of the main analysis. Panel A: Same data as in Figure 1 with the addition of the treatment group and the control group of the trial on enfortumab published by Powels et al. [18]. Panel B: Same data as in panel A but immunotherapies have been pooled into a single group. Endpoint, overall survival. Time expressed in months. Abbreviation: ICIs, immune checkpoint inhibitors.

Figure 2A shows the graph of the second set of KM curves that includes the above five treatments plus enfortumab vedotin. Furthermore, Figure 2B shows the same curves as in Figure 2A, but pembrolizumab, nivolumab, and atezolizumab have been grouped into a single arm denoted as immune checkpoint inhibitors (ICIs).

**Figure 3:**
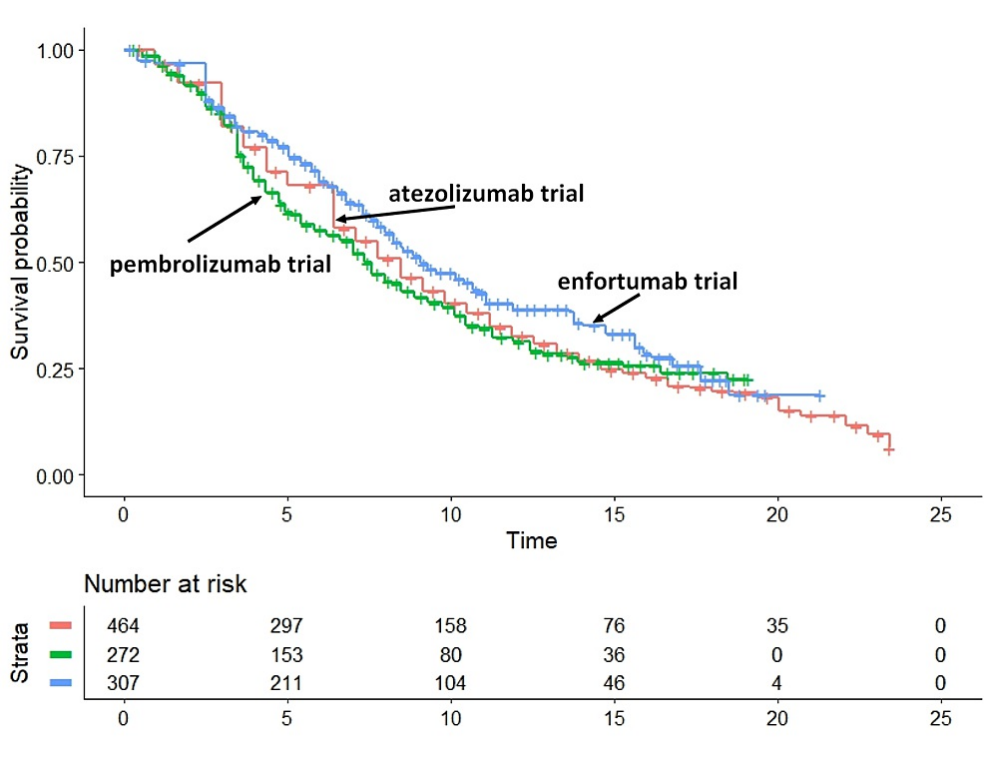
Heterogeneity test. This test that examined differences between the control groups of trials on pembrolizumab, atezolizumab, and enfortumab. These three control groups received standard chemotherapy. Endpoint, overall survival. Time expressed in months.

Based on this final dataset (Figure 2B), the head-to-head indirect comparisons gave the following results: (a) enfortumab versus chemotherapy: HR, 0.6645; 95%CI, 0.5571 to 0.7926 (p<0.001); hence, enfortumab fared significantly better than chemotherapy; (b) enfortumab versus ICIs: HR 0.748; 95%CI, 608 to 0.921 (p=0.0062); hence, enfortumab fared significantly better than ICIs, but only until 20 months when its curve crossed below that of ICIs. As expected, the other two indirect comparisons were less interesting. Their results were as follows: (a) ICIs versus chemotherapy: HR, 0.8882; 95%CI, 0.7950 to 0.9923 (p=0.0361); hence, ICIs fared significantly better than chemotherapy; (b) vinflunine versus chemotherapy: HR, 1.2019; 95%CI, 1.0279 to 1.4053 (p=0.0212); hence, vinflunine fared significantly worse than chemotherapy. Finally, Figure 3 shows the heterogeneity analysis involving the three control groups.

The head-to-head indirect comparisons between the three control groups gave the following results: (a) comparison of pembrolizumab versus atezolizumab trial controls: HR, 1.051; 95%CI, 0.8762 to 1.262 (p>0.05); (b) comparison of enfortumab versus atezolizumab trial controls: HR, 0.851; 95CI, 0.7103 to 1.020 (p>0.05); (c) comparison of enfortumab versus pembrolizumab trial controls: HR, 0.810; 95%CI, 0.626 to 1.047 (p>0.05). Interestingly, these data show that controls of the enfortumab trial fared slightly better than controls of the two ICI trials. This suggests that the remarkable survival results of enfortumab were, to a certain extent, influenced by the slightly better prognosis of the population enrolled in the enfortumab trial in comparison with patients enrolled in the two ICI trials. In summary, in the context of these four figures, Figure 2B is the one providing the most relevant message in terms of comparative effectiveness, but even Figure 3 must be taken into consideration.

## Discussion

Methods that reconstruct individual patient data have been extensively used both for estimating the RMST [15] and for performing indirect head-to-head comparisons [3-14]. Overall, nearly 30 topics have already been investigated since 2020, thus providing dozens of multi-trial datasets that summarize the current state of the art in terms of effectiveness. The experience described herein shows that, when a potential innovative treatment (enfortumab vedotin) is developed in an already investigated area (advanced urothelial cancer), the Shiny method can be applied according to the "one-to-many" approach. This allows us to quickly assess the place in therapy of the new treatment (the "one") and to evaluate whether the new treatment determines a relevant incremental benefit in comparison with previous treatments (the "many").

While these assessments are quick, their limitations must not be overlooked. The drawbacks of indirect comparisons have been extensively discussed in the previous literature [3-14], to which reference can be made. In brief, indirect comparisons lose the balancing effects due to randomization, and so a new treatment can perform better simply because the enrolled population had more favorable prognostic characteristics. In the present analysis, Figure 3 is an example of how this issue can be handled based on a post-hoc assessment of heterogeneity. Furthermore, one drawback of the present analysis is the assumption, not adequately proven, that the previous study by Rivano et al. [17] was representative of the main treatments available because a formal literature search was not conducted. The same problem might occur with other analyses belonging to the large group of Shiny studies previously published [3-15]. Finally, there could be potential issues regarding the ethical requirements of investigations conducted according to the Shiny method and therefore based, like the present one, on "reconstructed" patients. While studies based on reconstructed patient data represent a new type of scientific contribution, they, however, share the same ethical implications as systematic reviews and meta-analyses; for example, approval by an ethics committee is not required. It should be kept in mind that reconstructed patients are actually simulated patients; this is the main reason why Shiny studies are exempted from these requirements.

This article is a further example of the Shiny method, which has been implemented herein according to the new "one-to-many" design. The new implementation serves as an identifier of the new treatment's potential superiority over currently available options. While this is quite an interesting methodological advancement, on the other hand, the example of enfortumab vedotin in urothelial cancer is not only the first example of the new method but also an original finding itself, focused on a complex therapeutic area.

## Conclusions

Numerous studies have already documented that the Shiny method is a simple but efficient tool to summarize the effectiveness of multiple treatments proposed for the same clinical indication. Its main requirement is that all included studies have employed the same time-to-event endpoint (e.g., OS or PFS); furthermore, the Kaplan-Meier graph of the survival data must have been published. In the present report, an extension of the Shiny method has been described in which, after a previous analysis focused on multiple treatments, a new and potentially innovative agent is made available in the same therapeutic area. In this framework, the "one-to-many" approach of the Shiny method can be applied to assess how the new treatment compares with the previous ones.
